# Apelin and Vasopressin: The Yin and Yang of Water Balance

**DOI:** 10.3389/fendo.2021.735515

**Published:** 2021-11-22

**Authors:** Pierre-Emmanuel Girault-Sotias, Romain Gerbier, Adrien Flahault, Nadia de Mota, Catherine Llorens-Cortes

**Affiliations:** Laboratory of Central Neuropeptides in the Regulation of Body Fluid Homeostasis and Cardiovascular Functions, Center for Interdisciplinary Research in Biology (CIRB), French National Institute for Health and Medical Research (INSERM), Unit U1050, National Center for Scientific Research (CNRS), Mixed Research Unit 7241, College de France, Paris, France

**Keywords:** apelin, vasopressin, apelin receptor, metabolically stable apelin-17 analogs, diuresis, osmolality, hyponatremia

## Abstract

Apelin, a (neuro)vasoactive peptide, plays a prominent role in controlling body fluid homeostasis and cardiovascular functions. Experimental data performed in rodents have shown that apelin has an aquaretic effect *via* its central and renal actions. In the brain, apelin inhibits the phasic electrical activity of vasopressinergic neurons and the release of vasopressin from the posterior pituitary into the bloodstream and in the kidney, apelin regulates renal microcirculation and counteracts in the collecting duct, the antidiuretic effect of vasopressin occurring *via* the vasopressin receptor type 2. In humans and rodents, if plasma osmolality is increased by hypertonic saline infusion/water deprivation or decreased by water loading, plasma vasopressin and apelin are conversely regulated to maintain body fluid homeostasis. In patients with the syndrome of inappropriate antidiuresis, in which vasopressin hypersecretion leads to hyponatremia, the balance between apelin and vasopressin is significantly altered. In order to re-establish the correct balance, a metabolically stable apelin-17 analog, LIT01-196, was developed, to overcome the problem of the very short half-life (in the minute range) of apelin *in vivo.* In a rat experimental model of vasopressin-induced hyponatremia, subcutaneously (*s.c*.) administered LIT01-196 blocks the antidiuretic effect of vasopressin and the vasopressin-induced increase in urinary osmolality, and induces a progressive improvement in hyponatremia, suggesting that apelin receptor activation constitutes an original approach for hyponatremia treatment.

## 1 Discovery

The apelin story began in 1993 with the cloning of a cDNA for an orphan receptor, given the name “APJ receptor” (putative receptor protein related to the type 1 (AT1) angiotensin receptor) from a human genomic library (1). This seven-transmembrane domain G-protein coupled receptor (GPCR) displays 31% amino-acid (aa) sequence identity to the human AT1 receptor and is encoded by a gene on chromosome 11. However, it does not bind radiolabeled angiotensin II (Ang II) (1), and stimulation of the rat APJ receptor by Ang II does not modify cyclic adenosine monophosphate (cAMP) production, demonstrating that it is not an angiotensin receptor subtype (2). The gene encoding the APJ receptor has no introns in human and rodents (2–4). The human and the rat APJ receptors are 380 and 377 aa long, respectively. The APJ receptor aa sequence is conserved across species, with more than 90% sequence identity between human and rodents, and up to 50% sequence identity with other non-mammalian species, such as zebrafish and frog (2–5).

In 1998, the endogenous ligand of the orphan APJ receptor was isolated from bovine stomach tissue extracts (6). This 36-aa peptide was called apelin for **AP**J **E**ndogenous **LI**ga**N**d, and the APJ receptor was renamed the apelin receptor (ApelinR).

## 2 Synthesis and Metabolism of Apelin

### 2.1 Processing of Preproapelin

**Figure 1:**
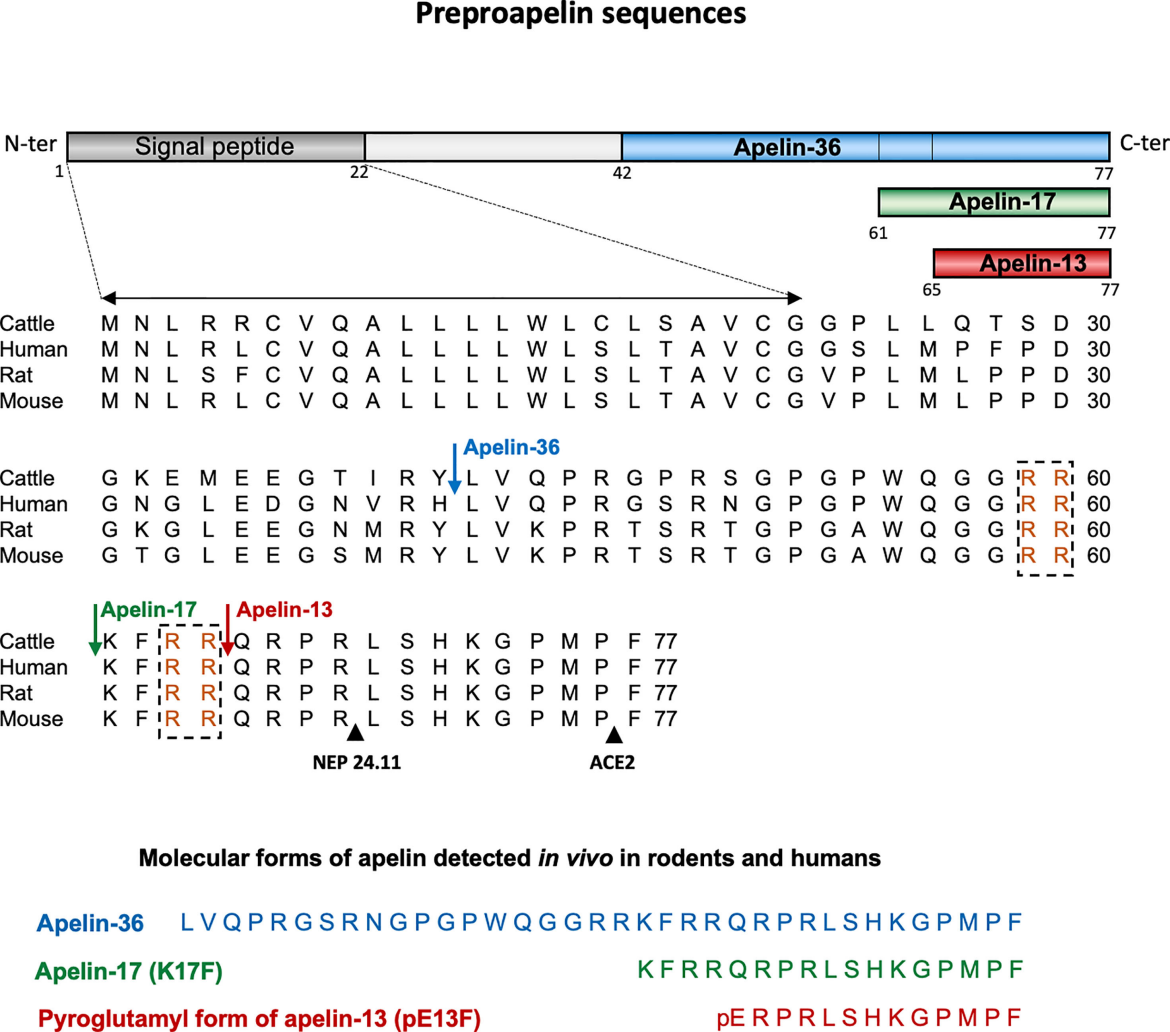
Amino-acid sequences of the apelin precursor, preproapelin, in cattle, humans, rats, and mice, and the molecular forms of apelin detected *in vivo*. The blue arrow indicates the beginning of the sequence of apelin-36, the green one that of the sequence of apelin-17 (K17F), which is strictly conserved in mammals and the red one that of the apelin-13 sequence. The dibasic doublets (in orange) are framed by black dashed boxes. The black arrows show the cleavage sites by neutral endopeptidase 24.11 (NEP 24.11, EC 3.4.24.11) and angiotensin-converting enzyme 2 (ACE-2, EC 3.4.17.23). The various molecular forms of apelin detected *in vivo* in mammals: apelin-36, apelin-17, and the pyroglutamyl form of apelin-13. Figure adapted from (7) with permission from the copyright holders.

Apelin is generated from a 77-aa precursor, preproapelin (**Figure 1**). The human apelin gene contains three exons, with the coding region spanning exons 1 and 2. The 3’ untranslated region also spans two exons (2 and 3) (8). This structure may account for the presence of transcripts of two different sizes (≈3 kb and ≈3.6 kb) in various tissues (3, 8). Alignment of the preproapelin aa sequences from cattle, humans, rats, and mice revealed strict conservation of the C-terminal 17 aa (aa 61 to 77 of the preproapelin sequence), known as apelin-17 or K17F (**Figure 1**). Various molecular forms of apelin, differing only in length, are present *in vivo* (36, 17, or 13 aa at the C-terminal part of preproapelin) commonly called apelin-36, apelin-17, and apelin-13. Apelin-13 is naturally pyroglutamylated at its N-terminus (pyroglutamyl form of apelin-13 or pE13F) (4, 9–12) (**Figure 1**).

Pairs of basic residues are present within the cattle, human, rat, and mouse preproapelin sequences, leading to the suggestion that prohormone convertases are responsible for processing the precursor to generate K17F and pE13F. The proprotein convertase subtilisin/kexin 3 (also named furin) has been shown to cleave *in vitro* proapelin directly into apelin-13 without generating longer isoforms (13).

For apelin-36 (amino acids 42 to 77 of the preproapelin sequence), the maturation mechanism remains unclear because there are no dibasic motifs upstream from the apelin-36 cleavage site.

Apelin-36 predominates in rat lung, testis, uterus, and in bovine colostrum, whereas both apelin-36 and pE13F have been detected in rat mammary gland (4, 10). The predominant forms of apelin in rat brain as well as in rat and human plasma are pE13F and K17F, with much lower concentrations of apelin-36 (11, 12). Apelin-13 is the most abundant form in the heart (14).

### 2.2 Metabolism of Apelin Peptides

The carboxypeptidase angiotensin-converting enzyme 2 (ACE-2, EC 3.4.17.23) removes the C-terminal phenylalanine residue of apelin-36, K17F or pE13F, both *in vitro* and *in vivo* (15, 16) **(** **Figure 1**). Moreover, it has recently been shown that neutral endopeptidase 24.11 or neprilysin (EC 3.4.24.11) hydrolyzes the scissile Arg**^8^-**Leu**^9^** and Arg**^4^-**Leu**^5^** peptide bonds of K17F and pE13F, respectively (**Figure 1**), generating two truncated peptides (17) unable to bind the ApelinR. NEP is, thus, the first protease shown to fully inactivate apelin. Synthetic analogs with the modified NEP degradation site (“RPRL” motif) have greater proteolytic stability *in vitro* while maintaining receptor affinities, highlighting the importance of this region for the full agonist activity of apelin (18).

## 3 Another Endogenous Ligand for the Apelin Receptor: Elabela/Apela

A second endogenous ligand of the ApelinR, apela (apelin receptor early endogenous ligand, also known as Elabela/Toddler (encoded by a gene on chromosome 4) was discovered in 2013 (19, 20). There is little sequence identity between apelin and apela, but both originate from precursors which are processed to generate several isoforms (21).

The gene for apela encoded a 54-amino acid precursor. The 22 aa signal peptide is removed to generate apela-32, which is secreted and bioactive (22, 23). The cleavage of apela-32 by furin to produce two fragments composed of 21 and 11 amino acids — apela-22 and apela-11, respectively — has been predicted (19, 20). The shortest C-terminal apela-11 fragment is fully conserved between species. Apela-32 and apela-22 display subnanomolar affinity for the ApelinR, whereas apela-11 is less active (22, 24). Apela is broadly expressed during development. In adults, apela mRNA levels are high in the prostate and kidney (25). In addition, the circulating apela in the bloodstream may originate at least partly from the endothelial cells of arterial vessels (22). For a review on apela see (21).

## 4 Pharmacological Characterization of the Apelin Receptor

The various molecular forms of apelin (apelin-36, K17F and pE13F) have subnanomolar affinities for the ApelinR (26, 27). Structure-function studies combining molecular modeling and site-directed mutagenesis have shown that the Arg^2^, Arg^4^, and Lys^8^ residues of pE13F interact with acidic aa residues of the ApelinR, located at its surface: Glu 172, Asp 282 and Asp 92, respectively (28).

Numerous studies have described the ApelinR signaling pathways activated by the different molecular forms of apelin. Apelin-36, K17F, and pE13F have similar abilities (in the subnanomolar range) to inhibit forskolin-induced cAMP production in Chinese Hamster Ovary (CHO) cells expressing the rat ApelinR and in Human Embryonic Kidney (HEK) cells expressing the human ApelinR (2, 9, 26, 29). Hosoya et al. showed that pertussis toxin blocked the inhibition of cAMP production induced by apelin-36 and pE13F, demonstrating the coupling of the ApelinR to Gα**_i_** (4). This finding was confirmed by Masri et al., who reported the preferential coupling of ApelinR to the Gα_i1_ and Gα_i2_ proteins (30, 31). Apelin-36, K17F, and pE13F also increase [Ca^2+^]_i_ mobilization in Ntera 2 human teratocarcinoma (NT2N) cells, in cells derived from basophils (RBL-2H3) and in HEK cells stably expressing the human ApelinR (26, 32–34). Morever, Hus-Citharel et al. showed that K17F decreases (AngII)-induced [Ca^2+^]_i_ mobilization in glomerular arterioles through the production of nitric oxide (NO) (35). Interestingly, several studies have shown that the stimulation of the ApelinR by apelin (K17F, pE13F) induces vasodilation and modulates vascular tone through NO production (35–38).

Activation of the apelin/APJ system can also induce a cascade of intracellular signaling kinases that regulate cell function. In human umbilical vein endothelial cells (HUVEC) and in CHO cells expressing the mouse ApelinR, activation of the apelin/APJ system induces activation of the phosphatidylinositol 3-kinase (PI3K)/Akt and the Extracellular Regulated Kinases (ERK1/2) pathways, stimulating phosphorylation of the S6 ribosomal protein kinase (p70S6K) (31, 39, 40). D’Aniello et al. showed that apelin induces phosphorylation of p70S6K in murine embryonic stem cells *via* an ERK1/2-dependent pathway (41). ERKs are phosphorylated in CHO cells stably expressing the mouse ApelinR in a Gα**_i_**-protein-dependent, protein kinase C (PKC)-dependent, and Ras-independent manner (30, 39).

Like most GPCRs, upon the binding of agonist ligands, the ApelinR elicits the recruitment of β-arrestins and their subsequent internalization through a clathrin-dependent mechanism (26, 27, 29, 34, 37, 42). Ser 348 at the C-terminus of the ApelinR has been identified as a crucial phosphorylation site for the interactions of this receptor with GRK2/5, β-arrestin1/2, and for its internalization (43). Furthermore, the C-terminal Phe residue of pE13F is embedded at the bottom of the binding site, in a hydrophobic cavity composed by Trp 152 in TMIV and Trp 259 and Phe 255 in TMVI (27).

Site-directed mutagenesis experiments revealed that Phe 255 and Trp 259, through their interactions with the C-terminal Phe residue of pE13F, were crucial for ApelinR internalization, but played no role in apelin binding or Gα**_i_** protein coupling. The C-terminal Phe residue of apelin is, thus, a key residue triggering ApelinR internalization (29, 44). Deletion of the C-terminal Phe residue of K17F (K16P) and the replacement of this residue with an alanine (K17A) strongly decrease the ability of the peptide to trigger ApelinR internalization, without affecting its affinity for the ApelinR or its ability to activate Gαi-coupling (16, 27, 29). All these data indicate functional dissociation between ApelinR G_i_-coupling and receptor internalization. This implies that the ApelinR exists in different active conformations, depending on the ligand fitting into the binding site, leading to the activation of different signaling pathways, and different subsequent biological effects (27). These findings suggest that ApelinR may display ‘functional selectivity’ or ‘biased signaling’, by coupling with G_i_ protein or recruiting β-arrestin 1 and 2. This hypothesis was confirmed by Ceraudo et al., who showed that K17F activates ERK1/2 in a β-arrestin-dependent and G_i_ protein-dependent manner, whereas K16P activates only the G_i_ protein (45). This functional selectivity of apelin peptides indicates that β-arrestin-dependent ERK1/2 activation, but not G_i_-dependent signaling, may contribute to the decrease in blood pressure (BP) induced by K17F. Indeed, when pE13A and K16P are injected intravenously in rats, they lost their capacity to decrease arterial BP when compared with the corresponding natural peptides, pE13F and K17F (29, 46). Moreover, the internalized ApelinR/pE13F complex is rapidly recycled to the cell surface through a Rab4-dependent mechanism whereas the internalized ApelinR/apelin-36 complex is targeted for degradation in lysosomes by Rab7 (47), showing that the trafficking of the ApelinR depends upon the ligand used to activate the receptor. These differences are consistent with studies showing that apelin-36 induces sustained, strong desensitization of the ApelinR, whereas the desensitization induced by pE13F is transient (30). The apelin isoforms therefore display subtle differences in pharmacological properties, which may influence their physiological actions.

Moreover, like many GPCRs, ApelinR may also form heterodimers *in vitro* with other GPCRs. ApelinR has been shown to dimerize with the AngII type 1 receptor (AT1R), leading to an inhibition of AngII signaling by apelin (48–50). ApelinR may also heterodimerize with the κ-opioid receptor, leading to an increase in cell proliferation through an increase in PKC activity and a decrease in protein kinase A activity (51). In HUVEC cells, ApelinR has been shown to heterodimerize with bradykinin type 1 receptor, leading to an increase in cell proliferation and the phosphorylation of eNOs through a G_q_ protein-dependent PKC signaling pathway (52).

## 5 Distribution of Apelin and Its Receptor

### 5.1 In the Brain

**Figure 2:**
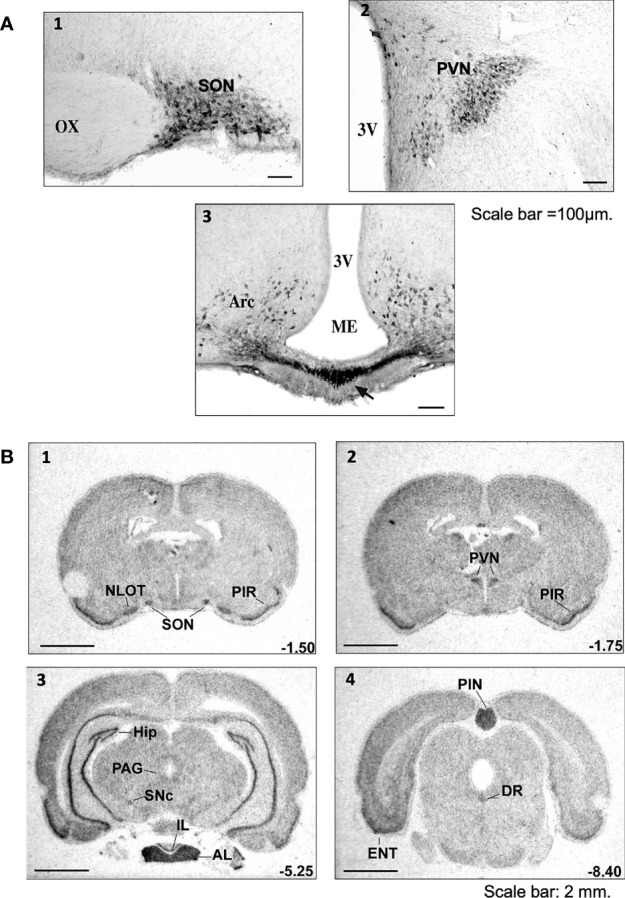
Distribution of apelinergic neurons and apelin-R mRNA in the adult rat brain **(A)** Coronal sections illustrating the distribution of apelin-containing cell bodies and nerve fibers in (1) the SON, (2) the PVN and (3) the Arc and ME of colchicine-treated adult rats. In the SON, the apelin-immunoreactive neurons and the nerve fibers are concentrated mostly in the ventral part of the nucleus. In the PVN, apelin-positive cell bodies and nerve fibers are found mostly in the magnocellular part of the nucleus. Numerous neuronal cell bodies were observed within the Arc, together with a higher density of nerve fibers in the internal layer of ME than in the external layer. Scale bar =100 µm. Figure adapted from (11, 54) with permission from the copyright holders. **(B)** Distribution of the rat ApelinR mRNA expression in the adult rat brain. The figures were scanned directly from the X-ray film. Representative frontal sections, at anteriorities determined from the bregma indicated in the lower right corner, from 1 to 4, were hybridized with the ApelinR antisense cRNA probe. Scale bar: 2 mm. Figure adapted from (11) with permission from the copyright holders. AL, anterior lobe of the pituitary gland; Arc, arcuate nucleus of the hypothalamus; DR, dorsal raphe nucleus; ENT, entorhinal cortex; HIP, hippocampus; IL, intermediate lobe of the pituitary gland; ME, median eminence; NLOT, nucleus of the lateral olfactory tract; OX, optic chiasma; PAG, periaqueductal gray matter; PIN, pineal gland; PIR, piriform cortex; PVN, paraventricular nucleus of the hypothalamus; SNc, pars compacta of the substantia nigra; SON, supraoptic nucleus; 3V, third ventricle.

Preproapelin is heterogeneously distributed between different brain structures (3, 8, 10, 26, 53). The distribution of apelinergic neurons in the adult rat brain has been studied using a polyclonal antibody with a high affinity and selectivity for K17F, which also recognizes pE13F and apelin-36 (11, 37, 54). Apelin-immunoreactive (IR) neuronal cell bodies are abundant in the hypothalamus and the medulla oblongata. These structures are involved in neuroendocrine control, food intake and the regulation of BP. They are abundant in the supraoptic nucleus (SON), the magnocellular part of the paraventricular nucleus (PVN), the arcuate nucleus, the nucleus ambiguus and the lateral reticular nucleus (54) (**Figure 2**). Conversely, the density of apelin-IR nerve fibers and nerve endings is high in the inner layer of the median eminence and in the posterior pituitary (37, 55), suggesting that, like magnocellular vasopressinergic and oxytocinergic neurons, the apelinergic neurons originating from the PVN and the SON project onto the posterior pituitary. Apelin was subsequently shown to colocalize with arginine-vasopressin (AVP) (11, 56) and oxytocin (55, 57) in magnocellular neurons. Apelin-IR cell bodies and fibers have also been identified in the subfornical organ (SFO), the organum vasculosum of the lamina terminalis (OVLT) and the median preoptic nucleus, all of which are involved in controlling drinking behavior (58, 59).

The ApelinR is also widely distributed in the rat central nervous system (CNS) (2, 3, 8). ApelinR mRNA has been identified in the piriform and entorhinal cortices, the hippocampus, the pars compacta of the substantia nigra, the dorsal raphe nucleus and the locus coeruleus (**Figure 2**). The last three of these structures are known to contain the neuronal cell bodies from dopaminergic, serotoninergic and noradrenergic neurons. High levels of apelinR mRNA have also been detected in the SON, PVN, arcuate nucleus, pineal gland and pituitary gland (2). Moreover, in the SON and PVN, the ApelinR (37, 60) and AVP receptor types 1a (V1a) and 1b (V1b), but not type 2 (V2-R) (61), are coexpressed by magnocellular AVP neurons. This finding provides strong evidence for the existence of an interaction between AVP and apelin.

### 5.2 In the Kidney

The mRNAs encoding preproapelin and ApelinR are expressed in rat and human kidney (3, 26). Apelin-like immunoreactivity has also been detected in human endothelial cells from small intrarenal vessels (62). Apelin expression has been detected in rat tubular epithelial cells, glomeruli and vascular epithelial cells (63), but another study reported restriction of apelin expression essentially to isolated cells in the medulla (64). An immunofluorescence study showed apelin to be present in the medullary collecting ducts (CD), with a distribution overlapping with that of aquaporin type 2 water channel (AQP2) (65).

ApelinR mRNA has been detected in the endothelial and vascular smooth muscle cells of rat glomerular arterioles (35). High levels of ApelinR mRNA are present in the glomeruli, reaching levels about eight times higher than those in nephron segments. Expression levels are moderate in all nephron segments (3, 35), including the collecting duct (CD), in which V2-R are also expressed (66). ApelinR mRNA levels are highest in the inner and outer stripes of the outer medulla (OM) and in the thick ascending limb (TAL) (35, 64, 65, 67).

## 6 Maintenance of Water Balance by Apelin and Vasopressin, Through Central and Renal Effects

### 6.1 Central Effects of Apelin on AVP Neuronal Activity, AVP Release and Diuresis

**Figure 3:**
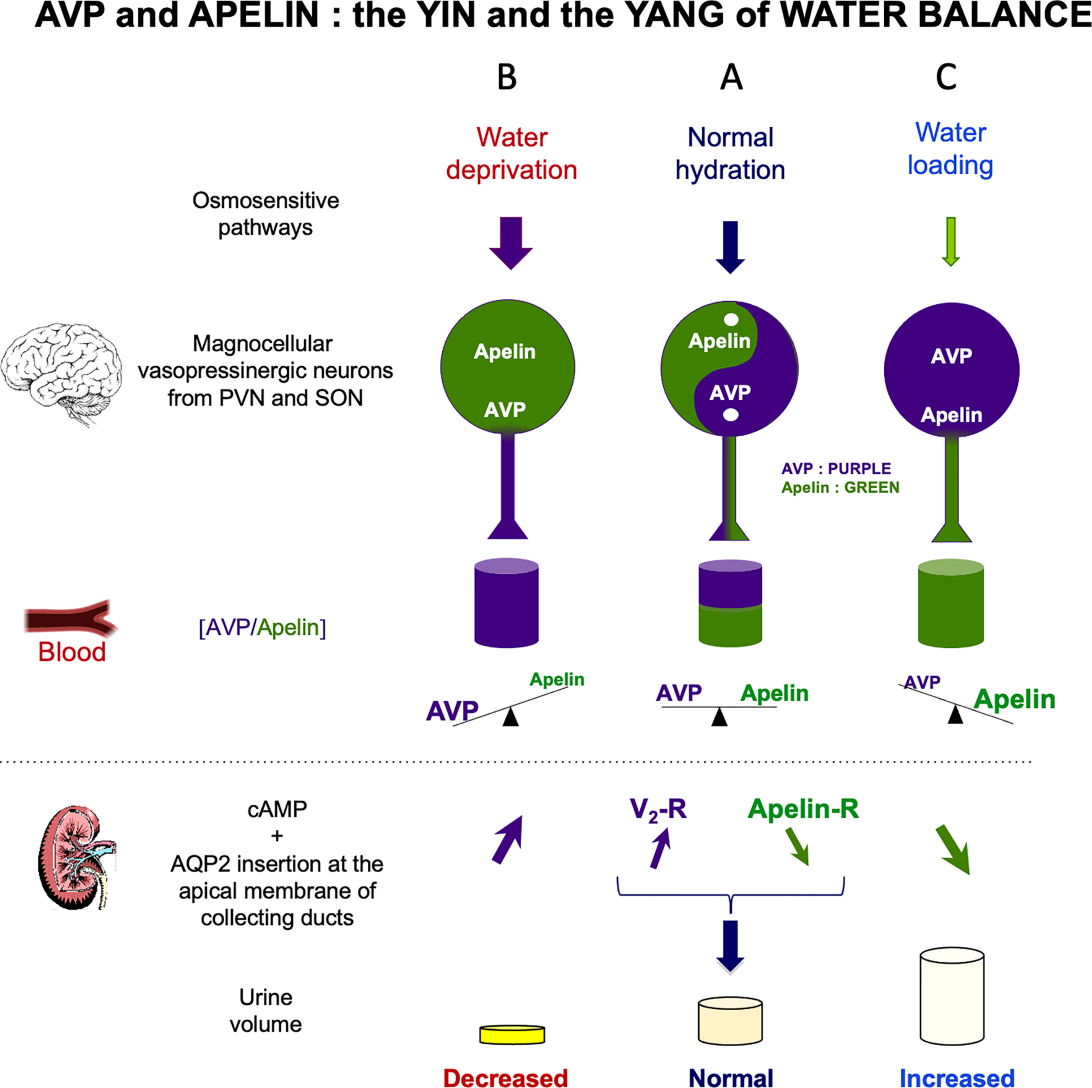
Vasopressin (AVP) and apelin: the yin and yang of water balance **(A)** In physiological conditions, apelin (green) and AVP (purple) are released in balanced proportions from the magnocellular AVP neurons, at levels appropriate for plasma osmolality. In the collecting duct of the kidney, AVP acts on V2-R to increase cAMP production and aquaporin-2 (AQP2) insertion into the apical membrane, leading to water reabsorption. Apelin has the opposite effect, through its action on the ApelinR. Water reabsorption is adequate in physiological conditions. **(B)** Following water deprivation in rodents: 1) AVP is released from magnocellular vasopressinergic neurons into the bloodstream more rapidly than it is synthesized, causing a depletion of AVP magnocellular neuronal content; 2) apelin release into the bloodstream decreases and apelin accumulates in magnocellular neurons. Thus, after dehydration, AVP and apelin are regulated in opposite manners, to facilitate systemic AVP release and suppress diuresis. **(C)** Following water loading in rodents: 1) AVP release is decreased from magnocellular vasopressinergic neurons into the bloodstream, causing an accumulation in AVP magnocellular neuronal content; 2) apelin release into the bloodstream increases, leading to a depletion of apelin magnocellular neuronal content. Thus, after water loading, AVP and apelin are regulated in opposite manners, to facilitate systemic apelin release and to increase aqueous diuresis. Figure adapted from (74) with permission from the copyright holders.

AVP, also known as antidiuretic hormone (ADH) is a peptide synthesized and released by hypothalamic magnocellular AVP neurons from the posterior pituitary into the bloodstream, in response to changes in plasma osmolality and volemia (68, 69) or under the influence of neurohormones, including natriuretic and angiotensin peptides (70, 71). The colocalization of AVP, apelin, V1 and apelin receptors in magnocellular neurons suggests an interaction between apelin and AVP. This raises the possibility of an effect of apelin in response to osmotic or volemic stimuli. This hypothesis was checked in two animal models. Studies were first performed in the lactating rat, which displays magnocellular AVP neuron hyperactivity, leading to an increase in AVP synthesis and release, to preserve water of the organism for an optimal milk production for the newborns (72, 73). In this model, the intracerebroventricular (*i.c.v.*) administration of apelin (K17F) (11) inhibits the phasic electrical activity of the magnocellular AVP neurons, reduces the release of AVP into the bloodstream and increases diuresis, without modifying sodium and potassium excretion (**Figure 3**). The second model used was mice deprived of water for 24/48 h, a condition known to increase AVP neuron activity and systemic AVP release (75, 76). In this model, *i.c.v.* K17F administration decreased systemic AVP release (11). These results suggest that apelin is probably released from the SON and PVN AVP cell bodies and inhibits AVP neuron activity and release through direct action on the apelin autoreceptors expressed by AVP/apelin-containing neurons. This mechanism probably involves apelin acting as a natural inhibitor of the antidiuretic effect of AVP.

On the other hand, in the anterior pituitary, apelin is highly co-expressed in corticotrophs and to a much lower extent in somatotrophs, and a high expression of ApelinR mRNA is also found in corticotrophs (77). Moreover, apelin was shown to act as a stimulatory autocrine/paracrine-acting peptide on adrenocorticotropic hormone (ACTH) release, suggesting a role for apelin in the regulation of the hypothalamo-pituitary adrenal (HPA) axis. Since ACTH at the adrenal level is a major stimulus of glucocorticoid secretion (78) and glucocorticoids were shown to increase water excretion possibly *via* an inhibition of AVP release (79), the aquaretic effect of apelin could also involve this pathway.

### 6.2 Renal Effects of Apelin on AVP-Induced Water Reabsorption

In addition to its central action, the aquaretic effect of apelin may involves a renal action, since apelin and its receptor are both expressed in the kidney (3, 26, 35, 62). Consistent with the presence of ApelinR mRNA in juxtamedullary efferent (EA) and afferent (AA) arterioles, the application of K17F on glomerular arterioles precontracted by AngII treatment induced NO-dependent vasorelaxation by inhibiting the Ang-II induced increase in intracellular calcium mobilization (35). This apelin-dependent vasorelaxation observed in the muscular EA, which give rise to the vasa recta, should result in an increase in renal blood flow, contributing to an increase in diuresis (35).

By stimulating V2-R in CD, AVP is known to induce an increase in cAMP production and to activate protein kinase A, which phosphorylates the AQP2. This results in the insertion of phosphorylated AQP2 into the apical membrane of the principal cells of the CD (80, 81), leading to water reabsorption, decreasing diuresis and plasma osmolality (**Figure 3**). The presence of ApelinR mRNA in the CD (35, 67) suggests that apelin could act as an aquaretic peptide through a direct action on this nephron segment. Consistent with this hypothesis, the intravenous injection of K17F in increasing doses in lactating rats or the continuous intravenous administration of apelin-13 administered for 24 h in alert male Sprague-Dawley alert rats (82) strongly increased diuresis in a dose-dependent manner, with a concomitant significant decrease in urine osmolality and no change in the excretion of Na^+^ and K^+^. Under these conditions, a significant decrease in apical AQP2 immunolabeling in the CD, with a corticomedullary gradient, was observed (83) (**Figure 3**). This finding is consistent with the inhibition, by K17F, in the medullary CD, of the cAMP production induced by (deamino-Cys^1^,D-Arg^8^)-vasopressin (dDAVP), a specific and selective V2-R agonist (83). These findings suggest that apelin may act as an aquaretic peptide through direct action on CD. Further evidence in support of this conclusion was recently provided by studies in a highly differentiated mouse cortical CD cell line (mpkCCD) expressing the V2-R and the ApelinR (84). The authors showed in this cell line that apelin-13 decreased the dDAVP-induced phosphorylation and apical membrane expression of AQP2 after 30–60 minutes of treatment, and decreased dDAVP-induced AQP2 mRNA and protein levels after 8–24 h of treatment (84). Furthermore, another study has shown that pE13F has diuretic effects potentially involving the cAMP/protein kinase A/soluble prorenin receptor pathway in the CD (85). Thus, the aquaretic effect of apelin is due not only to a central effect, inhibiting AVP release into the bloodstream, but also to a direct effect of apelin in the kidneys, increasing renal blood flow and counteracting the antidiuretic effect of AVP mediated *via* the V2-R in CD (**Figure 3**).

These results also show that apelin and AVP have opposite effects on the CD, contributing to the control of plasma osmolality by regulating water reabsorption by the kidney.

These results are consistent with those of other studies reporting an aquaretic role of apelin in rodents (82, 84–87). In addition, apelin gene expression in the brain has also been reported to be hydration-sensitive (88). It must also be taken into account that Elabela/apela which has the same affinity as apelin for the apelinR, has been shown to stimulate urine output and water intake in adult rats (82, 87) suggesting that Elabela/apela may therefore play with apelin a role in the regulation of body fluid homeostasis.

Studies on ApelinR^-/-^ mice (89, 90) have shown that water deprivation significantly decreases urine volume (by 61%) and increases urine osmolality (by 59%) in wild-type mice, with similar, but non-significant changes observed in ApelinR^-/-^ mice (-25%, and +26% respectively), suggesting that the ApelinR^-/-^ mice did not concentrate their urine to the same extent as wild-type mice. This effect was not related to an inability of ApelinR^-/-^ mice to increase their plasma AVP levels following water deprivation. In normal hydration conditions, plasma AVP levels in ApelinR^-/-^ mice (23.3 pg/ml) were 40% lower than those in wild-type mice (39.5 pg/ml). Following water deprivation, plasma AVP levels in ApelinR^-/-^ and wild-type mice were similar (52.9 and 57.7 pg/ml respectively). This showed that water deprivation increased plasma AVP levels by 127% in ApelinR^-/-^ mice whereas only by 46% in wild-type mice.

The authors also showed that treatment with dDAVP increased urinary osmolality more efficiently (+29%) in wild-type mice than in ApelinR^-/-^ mice. These observations suggest that the defect in water metabolism observed in ApelinR^-/-^ mice is not due to a decrease in plasma AVP levels but may result from a deficiency at the kidney level, like a decrease in the density of renal V2-R binding sites or in the signaling response of the V2R which remains to be investigated. These data are not in line with the aquaretic effect of apelin and apelin analogs (38, 82, 83, 86, 87, 91) but it cannot be excluded that the total absence of ApelinRs during fetal and adult life could elicit compensatory mechanisms, leading to these opposite effects on urine output and urine osmolality.

### 6.3 Opposite Patterns of Vasopressin and Apelin Regulation Following Water Deprivation

#### 6.3.1 In Rodents

The colocalization of apelin and AVP, and their opposite actions on diuresis raise questions about the regulation of these peptides to maintain body fluid homeostasis.

Following water deprivation in rodents, AVP is released into the bloodstream more rapidly that it is synthesized, causing a depletion of AVP neuronal content in magnocellular vasopressinergic neurons (92). In parallel, water deprivation decreases plasma apelin levels and induces an increase in apelin neuronal content in magnocellular vasopressinergic neurons (11, 56). Thus, following water deprivation, apelin accumulates in the vasopressinergic neurons rather than being released. This increase in apelin neuronal content observed in dehydrated rats is markedly reduced by the *i.c.v.* administration of a selective V1 receptor antagonist, whereas the *i.c.v.* infusion of AVP has effects on neuronal apelin concentration similar to those of dehydration, this effect being selectively blocked by the co-administration of a V1 receptor antagonist (56). The apelin and AVP responses to dehydration are, therefore, opposite (11, 92). These results imply that AVP and apelin are released separately by the magnocellular vasopressinergic neurons by which they are produced. Consistent with this hypothesis, double-immunolabeling confocal microscopy studies have shown that a large proportion of apelin immunoreactivity colocalizes with AVP in magnocellular neurons in the SON and the PVN, although these two peptides are found in different subcellular compartments (11, 56).

These studies show that the cross-regulation of apelin and AVP, in response to osmotic stimuli, has a physiological purpose: the maintenance of water balance in the body, through the prevention of water excretion by the kidney after water deprivation, and the promotion of water excretion after water loading.

#### 6.3.2 In Humans

Such cross-regulation of apelin and AVP in response to osmotic stimuli has also been studied in humans. The relationship between osmolality and plasma concentrations of apelin and AVP was investigated in healthy volunteers (12) after the infusion of hypertonic saline for 2 h to increase plasma osmolality or after 30 minutes of oral water loading to decrease plasma osmolality.

Increases in plasma osmolality were accompanied by a simultaneous increase in plasma AVP levels and a decrease in plasma apelin levels. Conversely, decreases in plasma osmolality led to lower plasma AVP levels and a rapid increase in plasma apelin levels (12). These observations are consistent with plasma osmolality acting as a major physiological regulator of plasma apelin levels in humans. Furthermore, the opposite patterns of apelin and AVP regulation by osmotic stimuli in humans are consistent with findings for rodents subjected to water deprivation. This strongly suggests that, like AVP, apelin participates in the maintenance of body fluid homeostasis in humans, as it does in rodents. Apelin and AVP can therefore be seen as the yin and yang of body fluid homeostasis.

## 7 The Apelin/AVP Balance and Hyponatremia

### 7.1 Hyponatremia

Hyponatremia, defined by a plasma sodium concentration below 135 mmol/l, is the most common electrolyte disorder in hospitalized patients. Various conditions have been associated with hyponatremia, including chronic heart failure, chronic kidney disease, liver cirrhosis, diuretic treatment and the Syndrome of Inappropriate Antidiuresis (SIAD), in which AVP secretion occurs in the absence of an osmotic or hemodynamic abnormality (93). It is important to recognize hyponatremia, because this condition is associated with high mortality rates (94–96) and can be a marker of underlying disease.

### 7.2 Syndrome of Inappropriate Antidiuresis

SIAD, previously known as the syndrome of inappropriate secretion of antidiuretic hormone (SIADH), is the most frequent cause of hyponatremia. Many clinical conditions may cause SIAD, including tumors, which may secrete AVP ectopically, central nervous system disorders and pulmonary diseases. SIAD may also result from the induction of increases in AVP secretion by various drugs, including tricyclic antidepressants, serotonin reuptake inhibitors and opiates, and/or from potentiation of the effects of AVP by drugs such as carbamazepine, chlorpropamide and non-steroidal anti-inflammatory drugs (97).

In SIAD, plasma AVP levels increase in a manner that is inappropriate relative to plasma osmolality (93). By acting on V2-R present in the CD of kidneys, the increased AVP levels stimulate cAMP production, leading to the insertion of AQP2 into the apical membrane of CD, resulting in higher levels of water reabsorption, lower levels of diuresis, and hyponatremia. Hyponatremia causes water entry into the cells due to the hypotonic state (98). Its symptoms result mostly from the enlargement of cells in the central nervous system, and their severity is dependent on serum sodium concentration. Severe symptoms, such as coma, convulsions, and respiratory arrest are usually associated with acute-onset severe hyponatremia. Less severe symptoms, such as headache, irritability, nausea/vomiting, mental slowing, and confusion, are observed in chronic hyponatremia (99).

Plasma apelin and AVP levels are regulated in opposite manners by osmotic stimuli in healthy subjects; this observation led to investigate the apelin response to the AVP osmoregulation defect in SIAD (100). In SIAD patients, sex- and age-adjusted plasma levels for apelin and copeptin (a biomarker of AVP release into the bloodstream in humans) are 26% and 75% higher, respectively, than those in healthy subjects (100). In 86% of SIAD patients, the plasma apelin/copeptin ratio lies outside the predicted range, highlighting the primary osmoregulatory defect in these patients. The abnormal apelin/AVP balance in hyponatremic SIAD patients may contribute to water retention (100). This has led to hypothesize that activation of the ApelinR with an ApelinR agonist might counteract AVP-induced water reabsorption, thereby correcting hyponatremia.

### 7.3 Effects of the Metabolically Stable Apelin-17 Analog LIT01-196 in an Experimental Model of Hyponatremia

#### 7.3.1 Development and Pharmacological Properties of LIT01-196

Endogenous apelin peptides have a short half-life *in vivo*. Gerbier et al. showed that K17F and pE13F have half-lives in mouse plasma of 4.6 and 7.2 minutes, respectively (38), and Murza et al. showed that pE13F has a half-life of 14 minutes in rat plasma (101). For apelin-36, Japp et al. suggested, based on experiments conducted in healthy human subjects, that the half-life of apelin-36 is less than five minutes (102). The half-life of K17F *in vivo* in the bloodstream after intravenous administration is 44 s in mice and 50 s in rats (86). These short half-lives result from the rapid metabolism of apelins by enzymes, such as ACE2 and NEP 24.11 (16, 17).

The short half-life of apelin *in vivo* has encouraged the development of metabolically stable apelin analogs for potential therapeutic applications. Numerous approaches (**Table 1**), such as PEGylation (107–109, 112, 113), synthetic modifications to the RPRL motif of apelin (18), palmitoylation and the use of unnatural amino acids (38, 103, 107, 114, 115), or main-chain modifications (cyclization) (106, 116, 117), have now been used to increase the half-life of apelin peptides. Recent studies have reported the development of nonpeptidic ApelinR agonists that mimic the signaling properties of apelin, some of them are orally active (**Table 1**) (104, 110, 111).

**Table 1:** Development of Apelin-R agonists.

	Affinity (Ki, nM)	cAMP production inhibition (IC_50_, nM)	β-arrestin (EC_50_, nM)	*Ex vivo*half-life in plasma (min)	*In vivo* half-life in bloodstream (min)	Diuresis
**PEPTIDIC ApelinR AGONISTS**						
**Apelin-13/pE13F and pE13F analogs**						
pE-R-P-R-L-S-H-K-G-P-M-P-F (38, 82, 101, 103, 104)	0.5	1.8	68 - 300	~ 10	<1 *after iv route*	**+** (82, 87, 91)
pE-R-P-R-L-S-H-K-G-P-Nle-P-F(L-αCH_3_) (101, 105)	0.3	0.07	–	> 120	–	–
MM07: Cyclo(1-6)C-R-P-R-L-C-H-K-G-P-M-P (106)	300	–	2130	–	17	–
pE-R-P-R-L-S-H-K-G-P-Nle-1Nal-D-α-Me-Y(OBn) (103)	0.08	3.8	36	438	26	–
**Apelin-17 (K17F) and K17F analogs**						
K17F: K-F-R-R-Q-R-P-R-L-S-H-K-G-P-M-P-F (38, 86)	0.06	0.30	15	4.6	<1 *after iv route*	**+** (83)
Fmoc-(PEG)_6_-NMeLeu-17A2 (107)	0.55	–	–	1620	–	–
P92: Ac-K-F-(D)R-R-(D)Q-R-P-R-(D)L-S-Aib-K-(D)A-P-Nle-P-4Br(F) (38)	0.09	0.56	4	24	–	++ (38)
LIT01-196: CF_3_(CF_2_)_7_(CH_2_)_2_C(O)-K-F-R-R-Q-R-P-R-L-S-H-K-G-P-M-P-F (38, 86)	0.08	1.71	16	> 1440	156 *after sc route*	++ (38, 86)
**Apelin-36 and apelin-36 analogs**						
Apelin-36: L-V-Q-P-R-G-S-R-N-G-P-G-P-W-Q-G-G-R-R-K-F-R-R-Q-R-P-R-L-S-H-K-G-P-M-P-F (4, 9, 46, 102)	2.4	0.5	–	–	< 5	–
40kDa-PEG-Apelin-36 (108, 109)	0.3	1.7	2	–	~ 20	–
**NON-PEPTIDIC ApelinR AGONISTS**						
CMF-019 (104)	2.6	0.1	224	38	–	–
AMG 986 (110)	–	0.23	0.25	–	144 *after iv route* *Orally active*	–
BMS-986224 (111)	0.07	0.02	7.9	–	*Orally active*	–

-, not determined; G, glycine; P, proline; A, alanine; V, valine; L, leucine; I, isoleucine; M, methionine; C, cysteine; F, phenylalanine; Y, tyrosine; W, tryptophan; H, histidine; K, lysine; R, arginine; Q, glutamine; N, asparagine; E, glutamic acid; D, aspartic acid; S, serine; T, threonine; Aib, aminoisobutyric acid; Y(OBn), tyrosine(Obenzyl); Nle, norleucine; 1Nal, 1-naphthylalanine; 4Br(F), 4-bromo-phenylalanine; PEG, polyethyleneglycol; Fmoc, 9-fluorenylmethyloxycarbonyl.

Most studies aiming to develop apelin analogs have focused on pE13F (38, 103, 105, 106, 115–117) and apelin-36 (108, 109, 112) (**Table 1**). However, K17F, which has an affinity 10 times higher than that of pE13F for human ApelinR, induces β-arrestin recruitment and the internalization of the rat ApelinR 10 to 30 times more strongly than pE13F, and also decreases arterial BP more effectively (29, 38).

**Figure 4:**
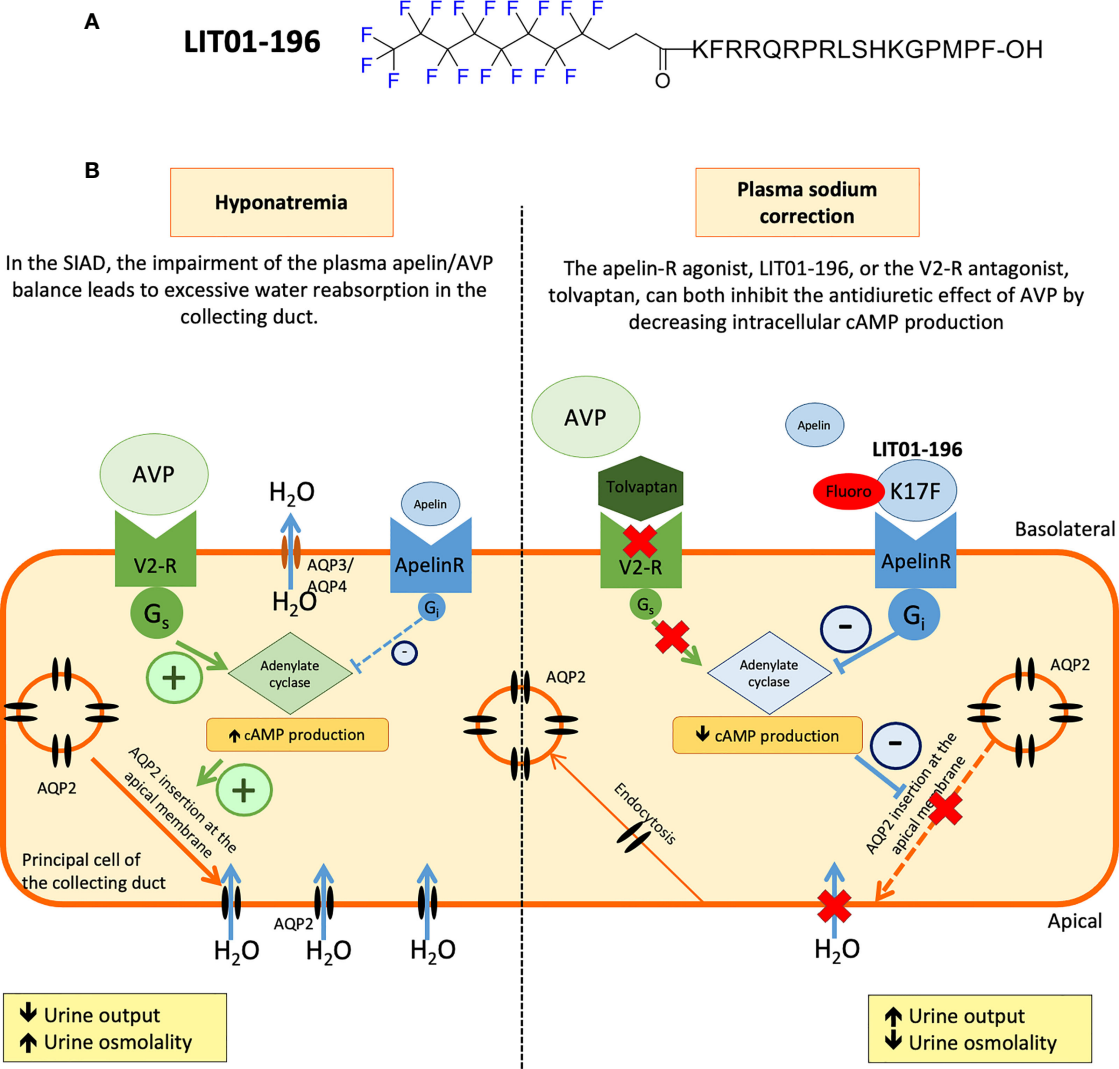
Proposed model of the effects of LIT01-196 on the principal cells of the collecting duct in SIAD. **(A)** Chemical structure of LIT01-196. **(B)** Schematic representation of the apelin and vasopressin (AVP). **(B)** Schematic representation of the apelin and vasopressin (AVP) receptors signaling pathways in the principal cells of the collecting duct (CD). Signaling pathways in the principal cells of the collecting duct (CD). In SIAD, the increase in AVP secretion is inadequate relative to plasma osmolality leading to hyponatremia. Consequently, there is an abnormal apelin/AVP balance in hyponatremic SIAD patients which contributes to water retention. By acting on V2 receptors (V2-R) present in the principal cells of the CD, the increased AVP levels stimulate cAMP production, leading to the insertion of aquaporin 2 (AQP2) into the apical membrane of CD, resulting in higher levels of water reabsorption (H_2_O), lower levels of diuresis, and hyponatremia. In SIAD, LIT01-196, by acting on the ApelinR present in the CD, re-establishes the “AVP/(apelin + LIT01-196)” balance and inhibits AVP-induced cAMP production, thereby inhibiting the insertion of AQP2 into the apical membrane of the CD, resulting in the inhibition of water reabsorption by the kidney and an increase in aqueous diuresis. As previously described, tolvaptan, by blocking the action of AVP on the V2-R, induces a similar increase in aqueous diuresis. Figure adapted from (86) with permission from the copyright holders.

Following these findings, metabolically stable K17F analogs have recently been developed (38, 107). Gerbier et al. used an original strategy for improving the protection of endogenous peptides against enzymatic degradation, based on the introduction of a fluorocarbon chain (FC) directly into the N-terminal part of K17F, generating LIT01-196 (**Figure 4**). This compound has a high affinity for the ApelinR (*K_i_* = 0.08 nM) and is much more stable in plasma (half-life >24 h) than K17F (4.6 min). LIT01-196 is remarkably resistant to plasma degrading-enzymes, with >90% of the peptide remaining unmodified after 24 h of incubation with mouse plasma at 37°C. LIT01-196 displays full agonist activity for cAMP production, ERK1/2 phosphorylation (nanomolar range), β-arrestin recruitment and the induction of ApelinR internalization (subnanomolar range) (38). Moreover, LIT01-196 has an *in vivo* half-life of 28 min in the bloodstream (*versus* 50 sec for K17F) after intravenous administration and 156 min after *s.c.* administration in alert control rats and was shown not to enter the brain after *s.c.* administration (38). The increase of the *in vivo* half-life of LIT01-196 in the blood circulation is probably due to the 69% binding of LIT01-196 to plasma proteins leading to the protection from enzymatic degradation and the reduction of renal clearance (38).

#### 7.3.2 Effects of LIT01-196 on AVP Release and AVP-Induced Antidiuresis in Control Animals

The central administration of LIT01-196 significantly decreases dehydration-induced systemic AVP release, and is 160 times more effective than K17F (38). These data suggest that LIT01-196 after *i.c.v* injection, like K17F, rapidly reaches the hypothalamic structures, the PVN and the SON, to stimulate the ApelinR expressed by AVP neurons, inhibit AVP release into the bloodstream and increase diuresis.

The effects of LIT01-196 were then investigated at the kidney level. First, *in vitro* in mpkCCD cells, LIT01-196 decreases dDAVP-induced cAMP production and apical cell surface expression of phosphorylated AQP2. These data suggested that systemic LIT01-196 administration in rats could induce like K17F an increase in aqueous diuresis. Indeed, LIT01-196 and tolvaptan (a V2-R) used as a reference aquaretic agent, administered at an equimolar dose of 900 nmol/kg by *s.c.* route, increase 24 h urine output similarly, by 79% and 77%, respectively, and decrease urinary osmolality by 52% and 40%, respectively, in control rats with normal natremia (86). This increase in urine output is associated with a significant increase in water intake in the tolvaptan group (+37%) and a slight increase in water intake in the LIT01-196 group (+11%). The sodium excretion fraction is unaffected by the administration of LIT01-196 or tolvaptan (86). These data suggest that LIT01-196 inhibits AVP-induced cAMP production in the CD, thereby inhibiting the insertion of AQP2 into the apical membrane, inhibiting water reabsorption by the kidney and increasing aqueous diuresis (**Figure 4**). Moreover, repeated *s.c.* administrations of LIT01-196 are not associated with renal failure or histological alterations of the kidney, and no change in plasma sodium, potassium, and glucose levels are observed in control rats (86).

On the other hand, LIT01-196 induces, like K17F, a vasorelaxation of the rat juxtamedullary arterioles that give rise to the vasa recta (38), suggesting that LIT01-196 can, like K17F, increase medullary blood flow and, therefore, diuresis (35). Thus, through its central and renal effects, LIT01-196 appears to be an effective aqueous diuretic of potential value for the treatment of water retention and hyponatremia.

#### 7.3.3 Effects of LIT01-196 on AVP-Induced Antidiuresis in an Experimental Model of Hyponatremia

In rats, the continuous *s.c.* infusion of AVP (30 ng/h) for four days, together with a semi-liquid diet, led to a decrease in urine output, an increase in urine osmolality and a decrease in plasma sodium levels, which stabilized at about 100 mmol/l for two to four days after the initiation of infusion.

The administration of LIT01-196 (900 nmol/kg, *s.c.*) for two days in this rat model of hyponatremia, by re-establishing the “AVP/(apelin + LIT01-196)” balance, inhibited the effects of AVP on urine output and urine osmolality effectively, and induced a progressive correction of plasma sodium levels (86) (**Figure 4**). In addition, chronic treatment with LIT01-196 is not associated with renal failure or histological alterations of the kidney, and no change in plasma sodium, potassium, and glucose levels is observed in control rats.

As expected from previous work (118), tolvaptan at the same equimolar dose (900 nmol/kg) also inhibits the antidiuretic effect of AVP. However, increasing the dose of tolvaptan results in an even larger increase in urine output, whereas increasing the dose of LIT01-196 did not. It can, therefore, be hypothesized that activating the ApelinR with a metabolically stable apelin analog, thereby re-establishing the apelin/AVP balance in the CD, rather than blocking the effects of AVP with V2-R antagonists, may result in less severe polyuria and may be better tolerated than V2-R antagonists. Moreover treatment by metabolically stable apelin analogs, may be also useful for the treatment of autosomal dominant polycystic kidney disease (ADPKD) (119) a disorder linked to excessive AVP secretion, since chronic treatment with tolvaptan, although effective, may be associated with intense thirst, polyuria (24 h diuresis around 6 L/day), nocturia (120) and a rapid plasma sodium concentration correction (121, 122). A too rapid plasma sodium correction in chronic hyponatremia can lead to osmotic demyelination, a severe neurological complication (123). Another situation where the apelin analogs would be useful in hyponatremic patients with hepatic insufficiency in whom the use of V2-R antagonists is contraindicated, due to their long-term hepatotoxic effect (124, 125).

Another application for metabolically stable apelin analogs could be in the nephrogenic syndrome of inappropriate antidiuresis (NSIAD). Gain-of-function mutations of the V2R are responsible for NSIAD. Patients with NSIAD have reduced free water excretion and concentrated urine despite hyponatremia and low or undetectable circulating AVP levels (126). This was due to substitution in the V2R sequence, of the arginine residue in position 137 by either a leucine or a cysteine (R137L/C) or the phenylalanine in position 229 by a valine (F229V) (127, 128). R137C was found to be the most frequent mutation. In absence of AVP, both R137L and R137C mutants displayed constitutive cAMP production and a high rate of arrestin-dependent constitutive internalization (128, 129). *In vitro* studies have shown that tolvaptan and satavaptan do not reduce constitutive increase of cAMP levels in R137L/C variants (130, 131). In line with *in vitro* data, a patient carrying the R137L did not respond to the administration of these V2R antagonists (132). The use of a metabolically stable apelin analog in the management of NSIAD could be of potential therapeutic interest since by activating the ApelinR, it will reduces cAMP production, reducing the insertion of AQP-2 at the apical membrane of collecting duct cells, thus decreasing water reabsorption and increasing urine output (84, 86).

This remains to be evaluated in further experimental studies and clinical trials. The various physiological effects associated with ApelinR activation result from the activation of different signaling pathways. The development of biased metabolically stable apelin-17 analogs targeting only the Gi signaling pathway might, therefore, increase their specificity of action for water metabolism.

## 8 Cardiovascular Actions of Apelin

Apelin has a wide range of physiological effects. Apelin plays a role in the cardiovascular system, acting on the endothelium of human mammary artery, human splanchnic arteries or rat glomerular arterioles and inducing vasodilation by increasing nitric oxide (NO) (35, 133, 134). In contrast, apelin exerts a direct vasoconstrictive effect on vascular smooth muscle cells in endothelium-denuded arteries (14, 135–137). Several *in vivo* studies have reported that different apelin analogs or apelinomimetics induce a rapid and dose-dependent reduction in BP, always mediated by NO (22, 29, 36, 46, 133, 138). This vasodilatory effect has also been observed in humans, where infusions of apelin-13 and apelin-36 result in a dose-dependent and NO-dependent arteriolar vasodilation in the forearm (106, 139). In healthy volunteers, apelin-13 induces a decrease in arterial BP, peripheral vascular resistance and induced a slight increase in heart rate which was probably a compensatory effect to the decrease in BP (102).

At the cardiac level, apelin is the most potent endogenous positive inotropic peptide discovered to date (140–142). Apelin reduces cardiac preload and afterload (143). Apelin also increases conduction velocity in cardiomyocytes and induces a shortening of action potential in atrial myocytes (144, 145). Apelin potently inhibited AngII-induced atrial fibrosis and subsequent vulnerability to atrial fibrillation induction (146). Administration of apelin or a small molecule apelinR agonist increases cardiac output *in vivo* in rodents (111, 147). Administration of apelin or apelin analogs in rodents post-myocardial infarction improved functional recovery and reduced infarct size, most likely due to increase NO production and angiogenesis (110, 148–150). Administration of apelin for 2 weeks after aortic banding prevented cardiac remodeling by inhibiting myocyte hypertrophy, cardiac fibrosis and ventricular dysfunction (151). In heart failure patients, acute administration of apelin, by intravenous route, increases cardiac output and left ventricular ejection fraction while reducing blood pressure and vascular resistance (102, 152). Apelin-knockout mice develop progressive impairment of cardiac contractility associated with systolic dysfunction in the absence of histological abnormalities. Importantly, pressure overload- induced heart failure is also more severe in apelin-deficient mice (153). Moreover, infusion of apelin using osmotic minipumps for 2 weeks in apelin-deficient mice restored the impaired cardiac function to that of wild-type mice (154).

Therefore, the use of ApelinR agonists may constitute a new therapeutic approach for the treatment of heart failure by increasing aqueous diuresis and cardiac contractility while decreasing vascular resistance.

## 9 Conclusion and Perspectives

The identification of apelin as the endogenous ligand of the orphan receptor APJ constituted an important step in basic research, with clinical implications. In animal models, experimental data have shown that the central injection of apelin into lactating rats inhibits the phasic electrical activity of AVP neurons, reduces plasma AVP levels, and increases aqueous diuresis. In the kidney, apelin increases aqueous diuresis by increasing renal blood flow and by counteracting the antidiuretic effect of AVP in the kidney at the tubular level. Following water deprivation or dehydration, in humans and rodents, AVP and apelin are conversely regulated, to facilitate systemic AVP release and to prevent additional water loss in the kidney. Moreover following water loading, AVP and apelin display an opposite pattern of regulation to facilitate systemic apelin release and increase aqueous diuresis to re-establish a water balance face to water overload. The available data show that AVP and apelin play a crucial role in maintaining body fluid homeostasis in humans and rodents. SIAD patients have an altered apelin-to-copeptin balance, contributing to the water metabolism defect. Apelin-R activation by a metabolically stable apelin-17 analog, LIT01-196, may constitute a promising therapeutic approach for the treatment of SIAD, by inhibiting the antidiuretic effect of AVP, increasing urine output, decreasing urine osmolality, moderately enhancing water intake, and progressively correcting hyponatremia.

## Author Notes

All appropriate permissions have been obtained from the copyright holders of **Figures 1**–**4**, which have been adapted and reproduced for this manuscript.

## Author Contributions

All authors listed have made substantial, direct, and intellectual contribution to the work and approved it for publication.

## Funding

These studies were supported by INSERM [Annual dotation], including the financial support for Proof of Concept, CoPoc Apelinatremia 2015–2017 from INSERM Transfert, the CNRS, the *Collège de France*, the *Agence Nationale de la Recherche* ANR-16-CE18-0030, FluoroPEP) and the *Federation Française de Cardiologie*. PEGS was supported by a fellowship from the *Fondation pour la Recherche Médicale*, grant number “PBR201810007643”. AF was supported by the fellowship from INSERM (Poste d’Accueil pour Hospitaliers).

## Conflict of Interest

The authors declare that the research was conducted in the absence of any commercial or financial relationships that could be construed as a potential conflict of interest.

## Publisher’s Note

All claims expressed in this article are solely those of the authors and do not necessarily represent those of their affiliated organizations, or those of the publisher, the editors and the reviewers. Any product that may be evaluated in this article, or claim that may be made by its manufacturer, is not guaranteed or endorsed by the publisher.
